# Simultaneous Measurement of the BOLD Effect and Metabolic Changes in Response to Visual Stimulation Using the MEGA-PRESS Sequence at 3 T

**DOI:** 10.3389/fnhum.2021.644079

**Published:** 2021-03-24

**Authors:** Gerard Eric Dwyer, Alexander R. Craven, Justyna Bereśniewicz, Katarzyna Kazimierczak, Lars Ersland, Kenneth Hugdahl, Renate Grüner

**Affiliations:** ^1^Department of Biological and Medical Psychology, University of Bergen, Bergen, Norway; ^2^NORMENT Centre of Excellence, Haukeland University Hospital, Bergen, Norway; ^3^Department of Clinical Engineering, Haukeland University Hospital, Bergen, Norway; ^4^Mohn Medical Imaging and Visualization Centre, Haukeland University Hospital, University of Bergen, Bergen, Norway; ^5^Department of Radiology, Haukeland University Hospital, Bergen, Norway; ^6^Division of Psychiatry, Haukeland University Hospital, Bergen, Norway; ^7^Department of Physics and Technology, University of Bergen, Bergen, Norway

**Keywords:** functional, spectroscopy, MRS, GABA, glutamate

## Abstract

The blood oxygen level dependent (BOLD) effect that provides the contrast in functional magnetic resonance imaging (fMRI) has been demonstrated to affect the linewidth of spectral peaks as measured with magnetic resonance spectroscopy (MRS) and through this, may be used as an indirect measure of cerebral blood flow related to neural activity. By acquiring MR-spectra interleaved with frames without water suppression, it may be possible to image the BOLD effect and associated metabolic changes simultaneously through changes in the linewidth of the unsuppressed water peak. The purpose of this study was to implement this approach with the MEGA-PRESS sequence, widely considered to be the standard sequence for quantitative measurement of GABA at field strengths of 3 T and lower, to observe how changes in both glutamate (measured as Glx) and GABA levels may relate to changes due to the BOLD effect. MR-spectra and fMRI were acquired from the occipital cortex (OCC) of 20 healthy participants whilst undergoing intrascanner visual stimulation in the form of a red and black radial checkerboard, alternating at 8 Hz, in 90 s blocks comprising 30 s of visual stimulation followed by 60 s of rest. Results show very strong agreement between the changes in the linewidth of the unsuppressed water signal and the canonical haemodynamic response function as well as a strong, negative, but not statistically significant, correlation with the Glx signal as measured from the OFF spectra in MEGA-PRESS pairs. Findings from this experiment suggest that the unsuppressed water signal provides a reliable measure of the BOLD effect and that correlations with associated changes in GABA and Glx levels may also be measured. However, discrepancies between metabolite levels as measured from the difference and OFF spectra raise questions regarding the reliability of the respective methods.

## Introduction

Performing magnetic resonance spectroscopy (MRS) in a time-resolved or functional manner makes it an ideal complement to functional magnetic resonance imaging (fMRI) in that it has the potential to allow patterns of neural activity to be related to associated biochemical events. Due to their roles as the principal excitatory and inhibitory neurotransmitters in the human brain, functional MRS studies have largely focused on dynamic changes in glutamate, or a composite signal of glutamate and glutamine denoted “Glx,” and γ-aminobutyric acid (GABA) levels. To date, functional spectroscopy paradigms have been used to measure increases in glutamate and lactate in the occipital cortex (OCC) in response to visual stimulation (Mangia et al., 2006, 2007; Lin et al., 2012; Schaller et al., 2013; Bednarík et al., 2015; Mekle et al., 2017; Boillat et al., 2019), changes in glutamate in the anterior cingulate cortex (ACC) and insula in response to pain (Mullins et al., 2005; Gussew et al., 2010; Gutzeit et al., 2011; Cleve et al., 2017) as well as dynamic changes in GABA in the sensorimotor cortex in response to learning (Floyer-Lea et al., 2006) and in the dorsolateral prefrontal cortex (DLPFC) under a working memory task (Michels et al., 2012).

Activity within a neural circuit may be characterized in terms of the balance of excitatory and inhibitory inputs to the circuit, commonly referred to as the excitation-inhibition balance (Denève and Machens, 2016; Jardri et al., 2016). As Isaacson and Scanziani (2011) illustrate, inhibition plays a critical role in shaping spontaneous and sensory-evoked cortical activity, placing a particular importance on the ability to quantify GABA in understanding the relationship between neural activity and the excitation-inhibition balance. Furthermore, where glutamate serves a myriad of functions in addition to its role as a neurotransmitter, including its roles in energy metabolism, protein synthesis and as a precursor to GABA (Agarwal and Renshaw, 2012; Mangia et al., 2012), in the brain GABA is almost exclusively a neurotransmitter, suggesting that changes in GABA levels as measured with MRS are likely to be more closely related to changes in the excitation-inhibition balance and synaptic transmission.

As challenging as it may be to investigate relationships between neural activity and glutamate or Glx, investigating relationships with GABA are further complicated by factors such as the relatively low biological concentration of GABA and significant spectral overlap with other more abundant metabolites. At lower field strengths (i.e., ≤ 3 T) many spectroscopy sequences may not sufficiently resolve GABA signals for accurate quantification (Gussew et al., 2010; Siniatchkin et al., 2012; Apšvalka et al., 2015). The MEGA-PRESS (MEscher-GArwood Point RESolved Spectroscopy) sequence (Mescher et al., 1998), widely considered to be the standard for performing MRS of GABA at field strengths of 3 T or less (Mullins et al., 2014), may facilitate accurate quantification of GABA, but requires the acquisition of two interleaved spectral datasets: one with a frequency selective editing pulse (“ON” spectrum) and one without (“OFF” spectrum) to create a difference spectrum, effectively reducing temporal resolution and complicating its implementation for functional paradigms.

Despite the usefulness of functional MRS, many implementations give no indication of neural activity or how it relates to changes in measured metabolite levels. Similarly, the blood oxygen level dependent (BOLD) effect that provides the contrast used in BOLD-fMRI provides a measure of changes in cerebral blood flow, which infers neural activity, but says little about its nature. However, the BOLD effect also induces a decrease in R${\;}_{2}^{*}$ rate, i.e., the inverse of T${\;}_{2}^{*}$, resulting in a decrease in linewidth and increase in height of spectral peaks (Just, 2020). Previous studies have utilized this phenomenon as an indirect measurement of the BOLD effect through changes in the linewidth of an unsuppressed water signal (Hennig et al., 1994; Frahm et al., 1996; Zhu and Chen, 2001). By interleaving spectral frames with and without water suppression, Apšvalka et al. (2015) demonstrated that it may be possible to exploit this effect to perform functional measurement of both the BOLD effect and related changes in Glx levels simultaneously.

The purpose of this study was to implement this approach with a GABA specific MEGA-PRESS sequence at 3 T, effectively permitting simultaneous functional imaging of the BOLD effect and changes in both Glx and GABA levels with the linewidth of the unsuppressed water signal as an indirect measure of the BOLD effect. MR-spectra were acquired from the occipital cortex (OCC) in response to visual stimulation in the form of a red-black radial checkerboard, alternating at a frequency of 8 Hz, a stimulation paradigm previously demonstrated to induce a measurable positive BOLD response (Kwong et al., 1992; Ogawa et al., 1992) and metabolic changes in the OCC (Mangia et al., 2006, 2007; Ip et al., 2017; Boillat et al., 2019). Previous studies suggest that visual stimulation will induce a measurable increase in Glx levels (Mangia et al., 2006, 2007; Ip et al., 2017; Boillat et al., 2019) and a possible decrease in GABA levels (Lin et al., 2012; Bednarík et al., 2015; Mekle et al., 2017). Assessment of activity through BOLD related linewidth changes predicts a significant difference in the linewidth of the unsuppressed water signal between spectra acquired during stimulation and at rest.

## Materials and Methods

This study was conducted under regional review board approved protocols (REK-Vest, REK case number 2016/1629) with written informed consent from all participants.

### Participants

The participant group for this study comprised 20 healthy individuals (mean age: 29 years, range: 20–40 years, 11 male). Based on self-report, participants were free from psychiatric and neurological conditions, and not currently using any psychoactive/psychotropic substances.

### MR-Imaging and Spectroscopy

All imaging and spectroscopy was performed on a 3 T GE 750 Discovery Scanner from GE Healthcare (General Electric, Milwaukee, United States of America) using a standard 8-channel head coil from Invivo (Invivo corp., Gainsville, Florida, United States of America).

**Figure 1:**
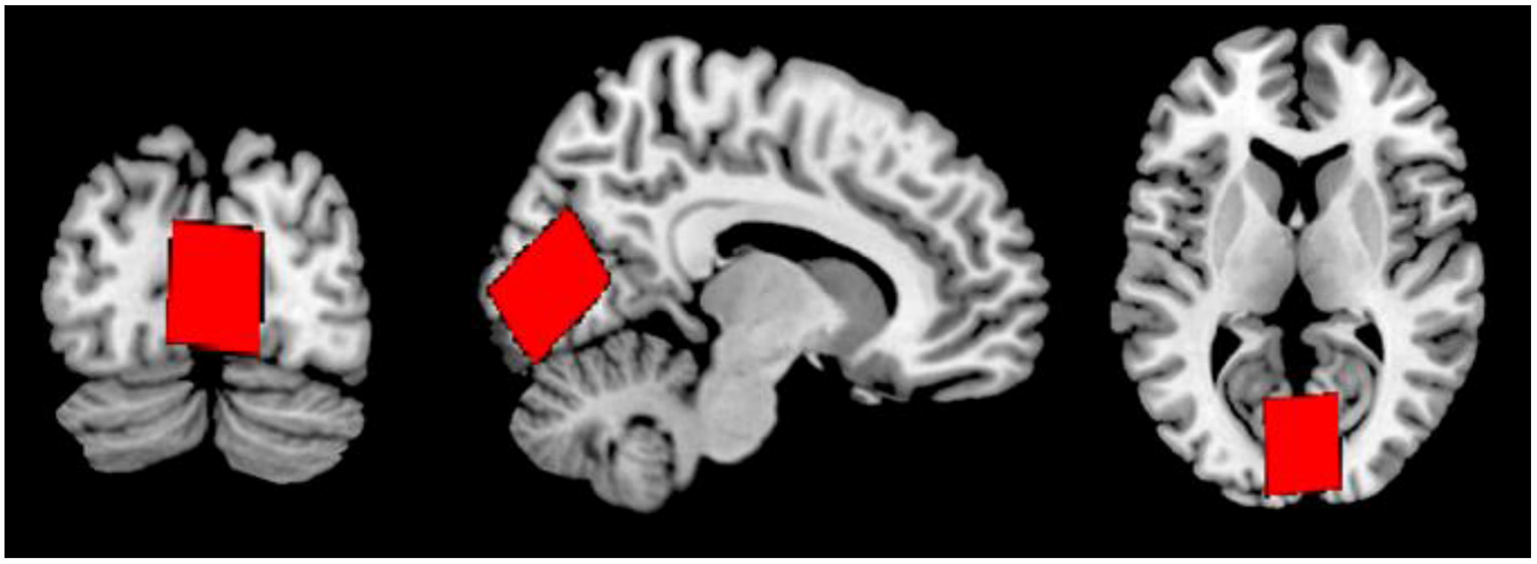
Voxel placement in the midline OCC in coronal (left), sagittal (middle), and axial (right) views.

Following a 3-plane localiser sequence (2D Spin Echo, TE = 80 ms, FOV = 240 mm, slice thickness = 8 mm, slice spacing = 15 mm) structural anatomical imaging was performed using a 3D T1 weighted fast spoiled gradient sequence (number of slices = 192, slice thickness = 1.0 mm, repetition time (TR) = 7.8 ms, echo time (TE) = 2.95 ms, field of view = 260 × 260 mm^2^, flip angle = 14 degrees, matrix = 256 × 256). These structural images were used to position a 31 × 26 × 24 mm^3^ voxel in the midline occipital cortex, across the longitudinal fissure and angled parallel to the parieto-occipital sulcus (Figure 1).

**Figure 2:**
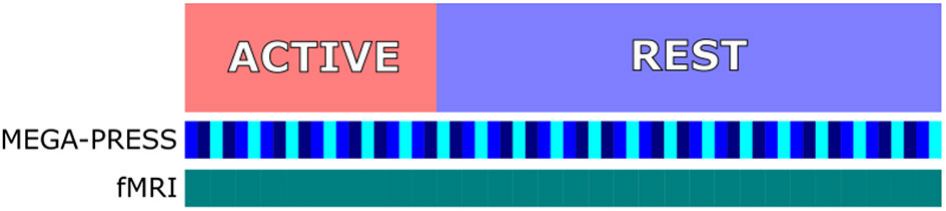
Experimental design showing one 90 s round of a 30 s active block (red) and 60 s rest block (blue). MEGA-PRESS spectra acquired with water suppression and editing pulse (“ON,” blue), with water suppression and without editing pulse (“OFF,” dark blue), and without water suppression or editing pulse (“REF” light blue). fMRI volumes acquired continuously with one volume acquired every 3 s (green).

Spectroscopy was performed using a GABA-specific MEGA-PRESS sequence (Mescher et al., 1998) (TE/TR = 68/1500 ms, editing pulses at 1.9 and 7.5 ppm) consisting of 600 spectral frames for a total acquisition time of 15 min and 30 s with an additional 16 frames acquired without water suppression to be used for scaling in quantitative metabolite estimates. Shimming, RF calibration and frequency adjustment were performed using an automated pre-scan prior to each spectral acquisition, providing a measure of the linewidth of the unsuppressed water signal that would be used in assessment of spectral quality. Spectra were acquired in groups of six spectral frames, first with water suppression and the MEGA-editing refocusing pulse (“ON”), secondly with water suppression and without the editing pulse (“OFF”), and thirdly without the editing pulse and without water suppression (“REF”), then with the ON and OFF spectra acquired in reverse order before the next reference frame (i.e., ON—OFF—REF—OFF—ON—REF repeated, Figure 2).

Following spectroscopy, BOLD-fMRI was performed using an echo-planar imaging (EPI) sequence (TR = 3000 ms, TE = 30 ms, image matrix = 96 × 96, FOV = 220 mm, flip angle = 90°, slice thickness = 3.0 mm, slice spacing 0.5 mm) with the same visual stimulation parameters, also for a total of 15 min and 30 s. In order to minimize the effects of thermal frequency drift on spectra, fMRI data were acquired after MRS for all participants.

**Figure 3:**
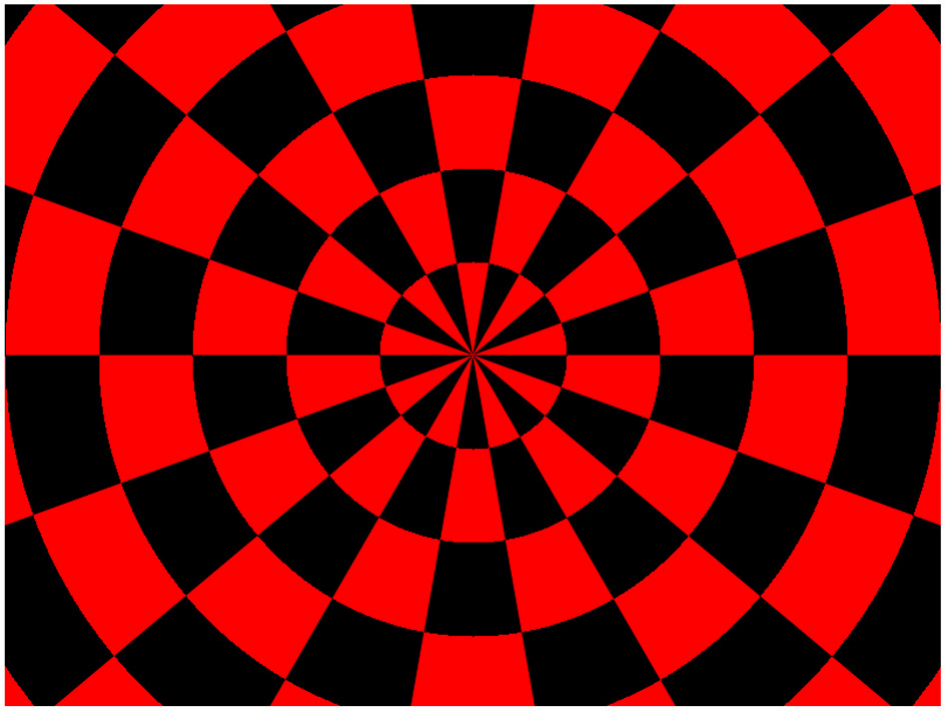
Radial red and black checkerboard used for visual stimulation.

Visual stimulation was delivered to participants through a set of MR-compatible binocular video goggles (NordicNeurolab Inc., Bergen, Norway) as an alternating, red-black, radial checkerboard (Figure 3) flickering at 8 Hz. Stimulation was delivered in blocks of 30 s followed by 1 min of a white fixation cross on black background, repeated for 8 blocks, with 2 min of fixation cross presented before the first and after the final stimulus presentation.

### Spectral Analysis

Following zero and first order phase correction, coil combination and frequency alignment, ON and OFF pairs were combined to produce MEGA-PRESS difference spectra providing quantitative estimates of Glx, GABA, and NAA. MEGA-PRESS OFF spectra were used to provide quantitative estimates of Glx, NAA, Creatine, Choline, lactate and glucose, equivalent to a PRESS sequence with TE = 68 ms. For each participant, four spectra were produced, a difference and OFF spectrum containing all frames acquired during visual stimulation blocks, referred to as the “Active” condition, and a difference and OFF spectrum containing all frames acquired as the white fixation cross was present, referred to as the “Rest” condition (Figure 2).

Quantitative spectral analysis was performed with LCModel (version 6.3-1J) (Provencher, 1993, 2001) using a simulated basis set (Dydak et al., 2011) with Kaiser coupling constants (Kaiser et al., 2008) to provide quantitative estimates of Glx, GABA and *N-*acetyl aspartate (NAA) from the MEGA-PRESS difference spectra, and from the OFF spectra in the MEGA-PRESS pairs: Glx (Glx OFF), NAA (NAA OFF), creatine (Cr OFF), choline (Cho OFF), lactate (Lac OFF), and glucose (Glc OFF).

It is important to note that when performing MEGA-edited GABA spectroscopy, co-edited macromolecule resonances contaminate the GABA signal. Thus, when referring to GABA as measured with a MEGA-PRESS sequence in this study, this refers to both GABA and the co-edited macromolecule (Edden et al., 2012).

### Unsuppressed Water Signal Analysis

In order to investigate how changes in the linewidth of the unsuppressed water signal relate to the haemodynamic response function (HRF), and how they may be used as a proxy measure of neural activity in the same manner as BOLD-fMRI, a response curve was constructed as a time course of the response for each stimulation block for each subject based on a method previously used by Brix et al. (2017) for investigating reproducibility of GABA measurements.

Linewidth was measured as the full-width at half-maximum (FWHM) in Hz. The time course was constructed using a Gaussian-weighted combination of between 60 and 100 time points of the FWHM of the unsuppressed water signal to produce a curve reflecting the change in FWHM of the unsuppressed water signal over the course of each 90 s stimulation block, including both active and rest periods.

Correlation was performed between the time courses for unsuppressed water FWHM and the task model convolved with a canonical haemodynamic response function (HRF) from the Statistical Parametrical Mapping software toolkit version 12 (SPM12, http://www.fil.ion.ucl.ac.uk/spm/).

### fMRI Analysis

Prior to analysis, all fMRI volumes acquired using the EPI sequence were converted from DICOM to NIfTI format using the dcm2nii program (http://people.cas.sc.edu/rorden/mricron/dcm2nii.html). Pre-processing of the converted images was performed using the Matlab/SPM based toolbox CONN (Whitfield-Gabrieli and Nieto-Castanon, 2012). Volumes were realigned to the first volume in each set and unwarped to correct for subject motion (Friston et al., 1995) then spatially normalized to an EPI template based on the Montreal Neurological Institute (MNI) standard reference brain (Evans et al., 1992). Images were finally smoothed through spatial convolution with a 5 mm Gaussian kernel.

Following pre-processing, beta values (i.e., parameter estimates in the general linear model) were extracted from a region of interest defined by a mask based on the placement of the spectroscopy voxel for each participant, using an in-house script drawing on the tools for NIfTI and ANALYZE image toolbox (https://se.mathworks.com/matlabcentral/fileexchange/8797-tools-for-nifti-and-analyze-image).

### Statistical Analyses

All statistical analyses were performed using R (R Development Core Team, 2020). Repeated measures *t*-tests were performed comparing the measured linewidth of the unsuppressed water signal (i.e., the measured difference, not subject to convolution with the HRF) and LCModel estimates for each of the measured metabolites between the rest and active conditions for each participant.

In order to investigate how changes in metabolite levels may relate both to one another and to changes in the local BOLD response, particularly with respect to the excitation/inhibition balance and Glx and GABA levels within the region (Isaacson and Scanziani, 2011), a correlation analysis was performed on the differences in all measured metabolite levels and the difference in unsuppressed water signal linewidth changes as measured (i.e., not subject to convolution with the HRF) between the active and rest blocks. Given the multiple comparisons performed as part of the correlation matrix, *p*-values for the correlation analysis were adjusted for using the false discovery rate (FDR) method (Benjamini and Hochberg, 1995; Benjamini and Yekutieli, 2001) as part of the p.adjust function in the R base package. Significance was tested at α = 0.05, results were considered statistically significant if the FDR adjusted *p*-value was < 0.05.

## Results

Due to poor quality data or withdrawal before fMRI data could be acquired, four participants were excluded from fMRI analyses. The resulting participant group for the fMRI analysis component comprised 16 individuals (mean age: 29 years, range: 20–40 years, 10 male).

**Figure 4:**
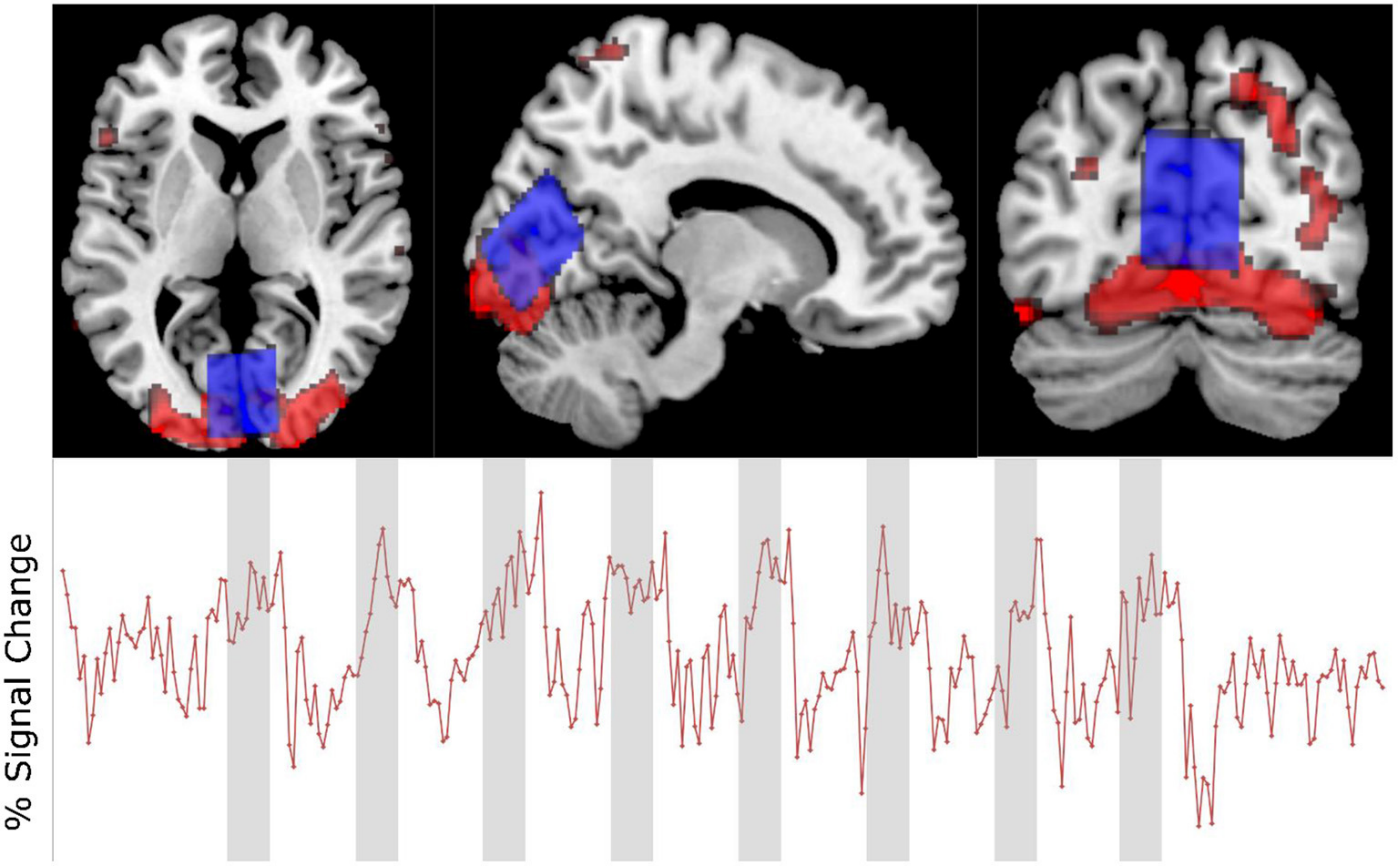
Upper: Voxel placement (blue) and activation map (red) in one participant. Lower: fMRI time course of same participant showing % signal change.

The fMRI data showed that the visual stimulation paradigm used elicited a positive BOLD response within the region of interest (Figure 4) with a mean difference in intensity between the active and rest blocks that was statistically significant [*t*(15) = 6.62, *p* < 0.001].

**Figure 5:**
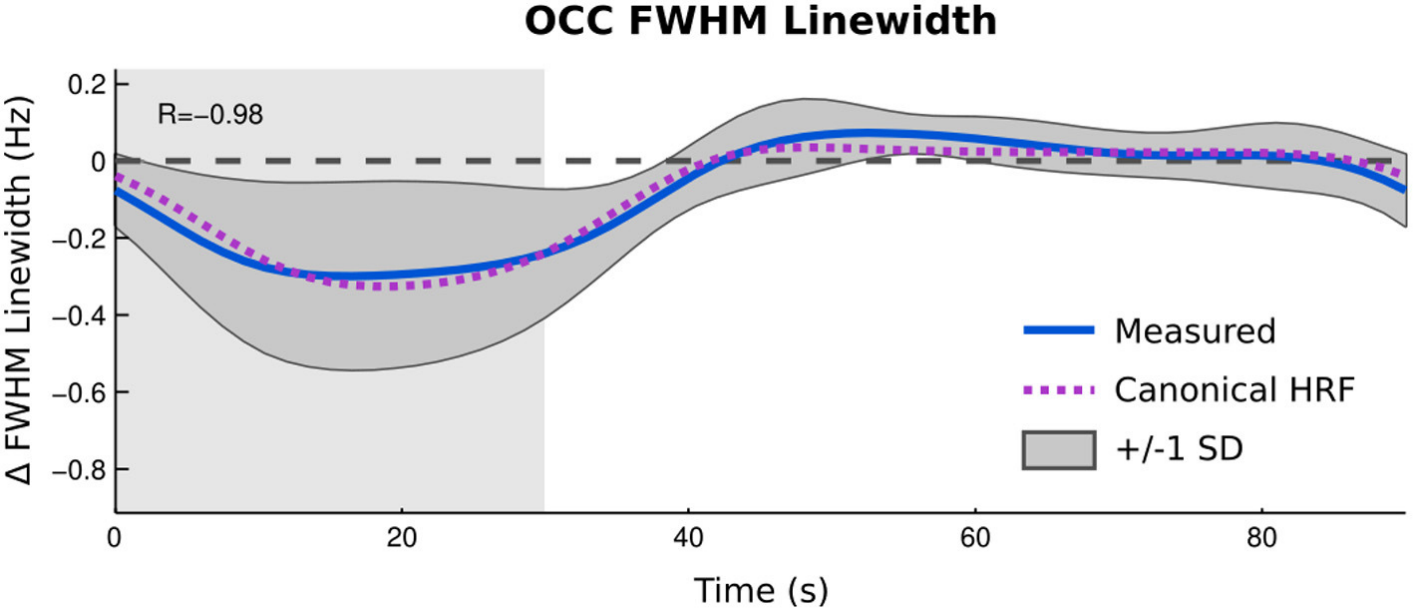
Time resolved analysis of changes in FWHM of unsuppressed water signal compared to predicted BOLD response between active blocks (gray background) and rest (white background). Zero line indicates average linewidth during rest blocks.

Figure 5 depicts the changes in the FWHM of the unsuppressed water signal reconstructed as a time-resolved curve. Linewidth changes in the unsuppressed water signal showed a very strong correlation between the group average of time courses for unsuppressed water FWHM and the task model convolved with a canonical haemodynamic response function (HRF) and the predicted haemodynamic response (*r* = −0.98, *p* < 0.001) with a mean change in FWHM between the active and rest blocks of 1.2%.

Prior to statistical analysis, spectral quality was assessed based on FWHM of the unsuppressed water peak measured during the automated prescan and Cramér-Rao lower bounds (%SD) for quantitative estimates provided by LCModel. Spectra with a prescan FWHM >12 Hz and %SD for GABA, Glx or Glx OFF great than or equal to 50 were excluded from further analyses. The resulting participant group for the MRS analysis comprised 11 individuals (mean age: 30 years, range: 21–40, seven male).

**Figure 6:**
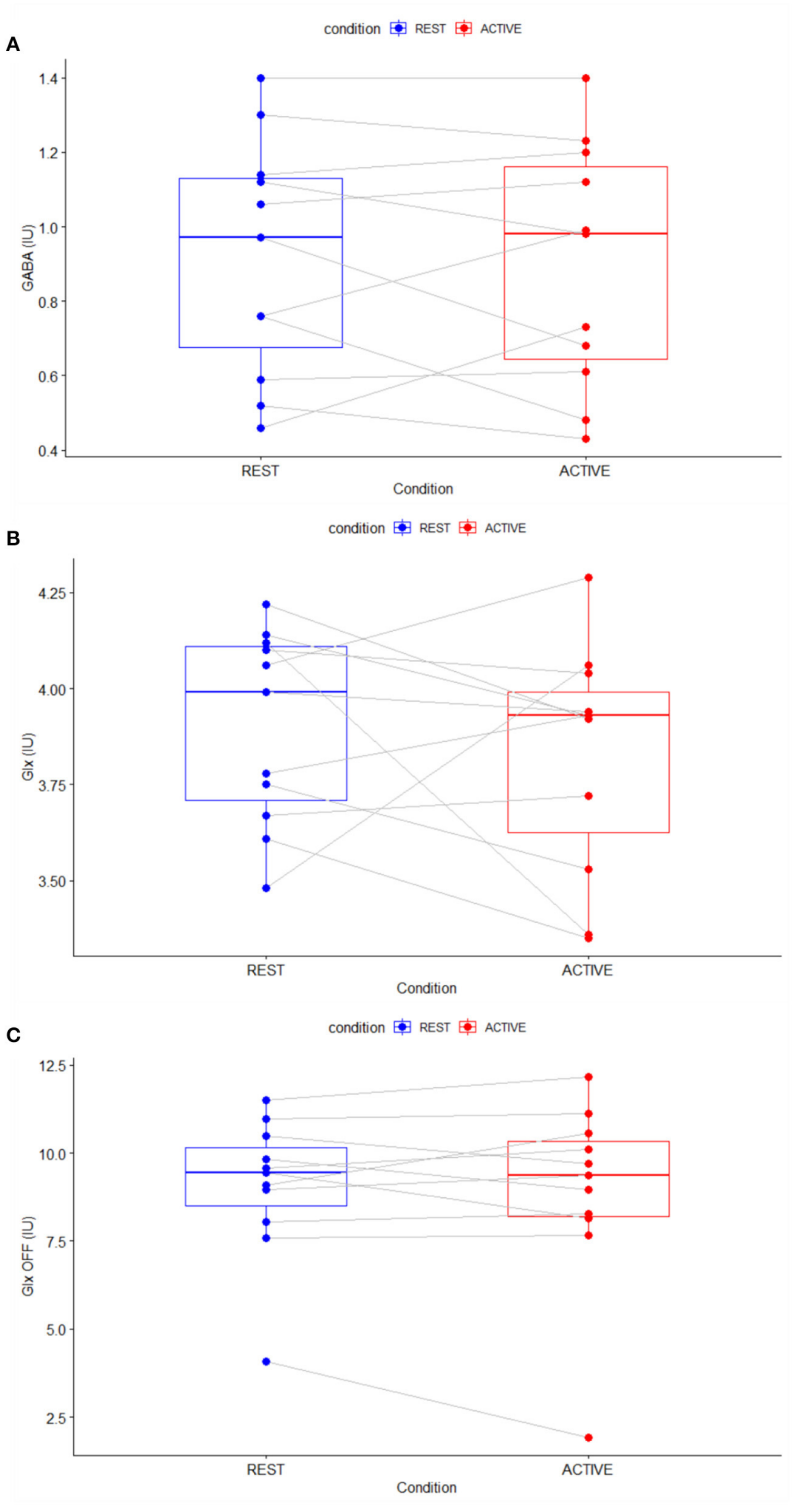
Parallel difference plots showing difference in metabolite levels between rest (blue) and active (red) conditions for GABA **(A)**, Glx **(B)**, and Glx measured from the OFF spectra **(C)**.

Repeated measures *t*-tests revealed no significant difference between the active and rest conditions for the linewidth of the unsuppressed water signal as it was measured [*t*(10) = 0.09, *p* = 0.92]. There were also no significant changes in GABA [*t*(10) = −0.39, *p* = 0.71] or Glx levels whether measured from the difference spectra [*t*(10) = −0.75, *p* = 0.47] or the OFF spectra [*t*(10) = −0.46, *p* = 0.66] (Figure 6). Differences were found in NAA levels as measured from the difference spectrum [*t*(10) = −1.43, *p* = 0.18] but did not reach statistical significance at the α = 0.05 level. Full results from the repeated measures *t*-tests are presented in Table 1.

**Table 1:** Metabolite estimates and Cramér-Rao lower bounds (%SD) for the active and rest conditions (*Mean, SD*) with results of repeated-measures *t*-tests.

**Metabolite**	**Active (IU)**	**Active %SD**	**Rest (IU)**	**Rest %SD**	**Mean of the difference**	***t***	***p***
GABA	0.89 (0.33)	18.27 (11.64)	0.92 (0.32)	18.18 (14.87)	−0.02	−0.39	0.71
Glx	3.82 (0.30)	8.36 (2.50)	3.90 (0.25)	7.45 (3.17)	−0.08	−0.75	0.47
NAA	6.03 (0.60)	1.64 (0.50)	6.23 (0.78)	1.36 (0.50)	−0.20	−1.43	0.18
Glx OFF	8.91 (2.68)	11.18 (11.91)	9.05 (2.02)	8.91 (4.46)	−0.14	−0.46	0.65
NAA OFF	14.22 (4.16)	2.00 (2.05)	14.14 (4.18)	1.91 (2.07)	0.08	0.77	0.45
Cr	9.08 (0.93)	4.71 (0.90)	8.98 (0.74)	4.67 (0.74)	0.09	1.22	0.25
Cho	1.53 (0.23)	1.84 (6.11)	1.51 (0.20)	0.45 (1.51)	0.02	0.92	0.38
Lac	0.67 (0.71)	304.64 (446.44)	0.31 (0.33)	282.00 (365.40)	0.36	1.53	0.16
Glc	2.79 (0.51)	19.45 (4.03)	2.66 (0.92)	22.00 (8.10)	0.13	0.76	0.46

**Figure 7:**
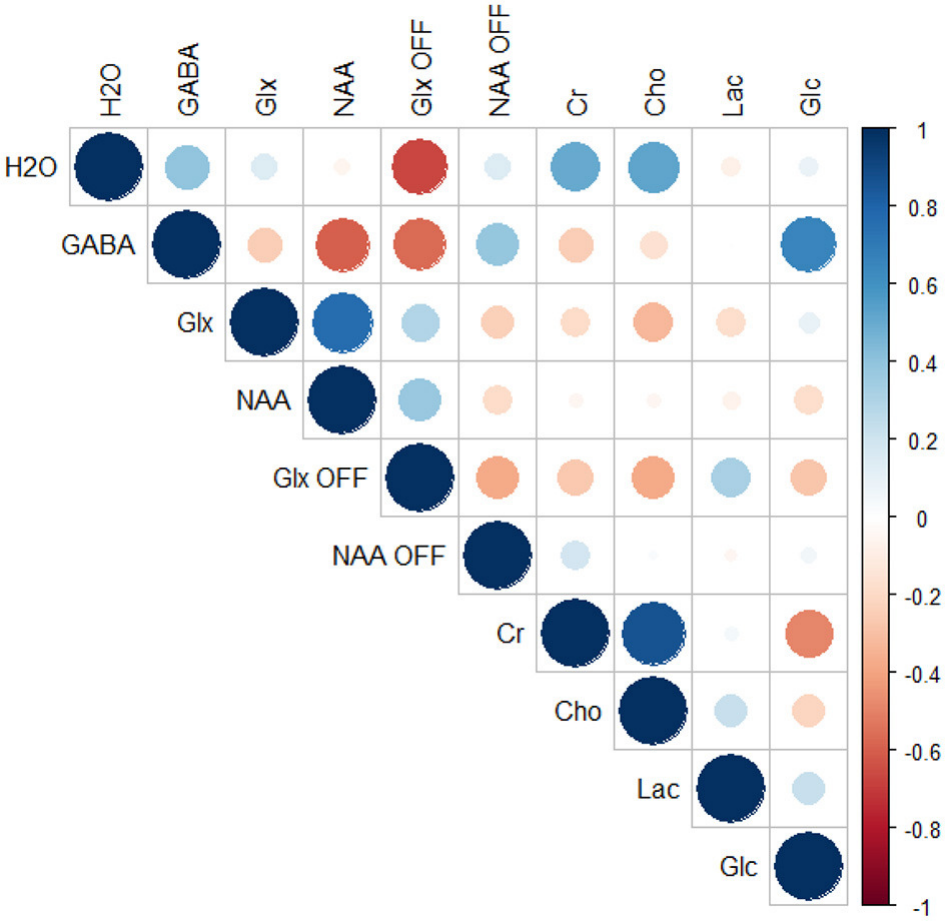
Correlation plot depicting differences in linewidth of unsuppressed water signal (H_2_O) and all measured metabolites.

Correlation analysis of the differences between the active and rest blocks revealed a moderate negative correlation between the change in the measured water peak linewidth and the change in Glx as measured from the OFF spectra (*r* = −0.66, *p* = 0.03, FDR adjusted *p* = 0.31) but not from the difference spectra (*r* = 0.14, *p* = 0.67, FDR adjusted *p* = 0.93). Correlations were also observed between GABA levels as measured from the difference spectra and NAA (*r* = −0.60, *p* = 0.05, FDR adjusted *p* = 0.48) and glucose (*r* = 0.66, *p* = 0.03, FDR adjusted *p* = 0.31), and a moderate negative correlation was observed between GABA and Glx as measured from the OFF spectra (*r* = −0.56, *p* = 0.07, FDR adjusted *p* = 0.54). However, none of these correlations were statistically significant when accounting for multiple comparisons. Full results from the correlation analysis are provided in Table 2 and depicted graphically in a correlation plot in Figure 7.

**Table 2:** Correlation matrix for differences between active and rest for linewidth of the unsuppressed water signal (H_2_O) and measured metabolites.

**Correlation (Pearson's r)**										
	**H**_**2**_**O**	**GABA**	**Glx**	**NAA**	**Glx OFF**	**NAA OFF**	**Cr**	**Cho**	**Lac**	**Glc**
H_2_O	1.00									
GABA	0.40	1.00								
Glx	0.14	−0.25	1.00							
NAA	−0.06	−0.60	0.76	1.00						
Glx OFF	−0.66	−0.56	0.29	0.37	1.00					
NAA OFF	0.15	0.39	−0.23	−0.19	−0.38	1.00				
Cr	0.51	−0.25	−0.19	−0.04	−0.27	0.18	1.00			
Cho	0.53	−0.15	−0.33	−0.05	−0.37	0.02	0.86	1.00		
Lac	−0.08	0.01	−0.17	−0.07	0.33	−0.04	0.05	0.22	1.00	
Glc	0.08	0.66	0.10	−0.18	−0.28	0.06	−0.48	−0.22	0.23	1.00
***p*****-values (lower) and FDR-adjusted** ***p*****-values (upper)**										
H_2_O		0.83	0.93	0.95	0.31	0.93	0.62	0.62	0.95	0.95
GABA	0.22		0.91	0.48	0.54	0.83	0.91	0.93	0.99	0.31
Glx	0.68	0.46		0.15	0.91	0.91	0.91	0.91	0.91	0.95
NAA	0.87	0.05	0.01		0.83	0.91	0.95	0.95	0.95	0.91
Glx OFF	0.03	0.07	0.38	0.26		0.83	0.91	0.83	0.91	0.91
NAA OFF	0.67	0.24	0.49	0.58	0.25		0.91	0.97	0.95	0.95
Cr	0.11	0.46	0.58	0.90	0.42	0.59		0.03	0.95	0.67
Cho	0.10	0.65	0.33	0.89	0.26	0.95	0.00		0.91	0.91
Lac	0.82	0.99	0.61	0.84	0.33	0.91	0.89	0.51		0.91
Glc	0.81	0.03	0.78	0.60	0.41	0.87	0.13	0.52	0.51	

## Discussion

The purpose of this study was to determine whether a MEGA-PRESS sequence, modified to include spectral frames without water suppression, could be used to perform simultaneous measurement of the BOLD effect and associated metabolic changes. This implementation is based on one previously used by Apšvalka et al. (2015) with a PRESS sequence (TE/TR = 105/1500 ms) for simultaneous measurement of the BOLD effect and glutamate dynamics in response to a repetition suppression paradigm. The advantage to this implementation with a MEGA-PRESS sequence is the ability to measure both GABA and glutamate (as Glx) dynamics at a field strength of 3 T. The results show that the unsuppressed water signal provides a reliable measure of the BOLD effect, and the experimental design and analysis methods used allow differences in the water signal linewidth and differences in metabolite levels to be assessed in terms of how they correlated with one another. However, this study also illustrates a number of problems regarding the use of spectral editing methods in functional spectroscopy paradigms.

One of the issues adversely affecting this this study was the amount of data that had to be excluded from analysis as part of the MRS component. Though 20 participants were scanned, nine were excluded from final analyses, mostly due to large errors surrounding metabolite estimates. Though small, this number is comparable to other, similar studies employing fMRS paradigms (e.g., *N* = 13 (Apšvalka et al., 2015), *N* = 13 (Kupers et al., 2009), *N* = 12 (Mullins et al., 2005). A power analysis conducted with G^*^power (Faul et al., 2009) found that the correlation analysis component of this study had sufficient power to detect a medium to strong effect (for *d* > 0.6, 1–β = 0.74, α = 0.05). However, for changes in Glx and GABA in the OCC in response to visual stimulation, the effect may be much smaller.

According to both the fMRI acquisition and the changes in the FWHM of the unsuppressed water signal, the visual stimulation paradigm used in this experiment was able to elicit a BOLD effect in the area of interest (Figure 4). The agreement between the changes in FWHM over the course of a 90 s experimental block and the modeled response show that the unsuppressed water signal provides a reliable measure of the BOLD effect (Figure 5), echoing the findings of Frahm et al. (1996) and Hennig et al. (1994) who, in the early days of fMRI suggested that it could be a useful alternative on systems where appropriate imaging sequences could not be implemented.

Despite this, measuring changes in spectral linewidth offers little advantage over conventional BOLD-fMRI in measuring haemodynamic responses. The advantage to this method was the ability to measure both the BOLD response and associated metabolic changes. The repeated measures *t*-tests revealed no significant changes between the rest and active conditions, and while the correlation analysis similarly revealed no statistically significant correlations when adjusted for multiple comparisons, a number of the finds warrant further discussion.

The observed correlation between the change in water signal linewidth and Glx OFF is consistent with many of the common findings from both fMRS and combined fMRI/MRS studies that glutamate or Glx levels correlate positively with a positive BOLD signal or a task/stimulus positive condition (Mullins et al., 2005; Mangia et al., 2007; Gussew et al., 2010; Lin et al., 2012; Apšvalka et al., 2015; Bednarík et al., 2015; Cleve et al., 2015; Ip et al., 2017). It is worth bearing in mind that the linewidth of the water signal is expected to decrease as a result of the BOLD effect, hence the negative correlation. What is interesting, however, is that the correlation was only observed in the Glx signal as measured from the MEGA-PRESS OFF spectrum, and not from the difference spectrum.

Although previous studies have found the MEGA-PRESS sequence to be comparable to standard short echo time PRESS sequences for quantitative estimates of the Glx signal (Henry et al., 2011), the presence of such a strong, positive correlation between Glx measured from the OFF spectra but not from the difference spectra raises questions regarding both the accuracy and sensitivity of the two approaches to quantifying Glx when using the MEGA-PRESS sequence. Maddock et al. (2018) compared the two measurements of the Glx signal, i.e., from the difference and OFF spectra from a MEGA-PRESS sequence (TE/TR = 68/1500 ms), with the Glx signal from a glutamate optimized PRESS sequence (TE/TR = 80/1500 ms) and found that in healthy participants, Glx measured from the OFF spectra correlated much more strongly (*r* ≥ 0.88) than the difference spectra (*r* ≤ 0.36) with the PRESS measurements. This discrepancy suggests that difference spectra may not be as sensitive to dynamic changes in metabolite levels as unedited spectra, but as there is no alternative measure for GABA in the OFF-spectra, there is insufficient evidence to determine how it applies to GABA in this study. It is possible that the reduced amount of spectral information available in the difference spectrum makes it less sensitive to dynamic changes.

In addition to being considered the optimal method for quantitative measures of GABA at field strengths of 3 T and lower, the findings of Mullins (2018) suggest that another benefit to measuring GABA with the MEGA-PRESS sequence, particularly in a functional capacity, may be the ability to distinguish synaptic GABA from vesicular GABA. Due to restrictions on their ability to tumble and move when packaged in synaptic vesicles, vesicular neurotransmitters may have a shorter T_2_ time than neurotransmitters in the cytosol or synapse. Using short echo time sequences (i.e., ≤ 15 ms) such as those frequently used with STEAM sequences and similar at 7 T, the measurement of neurotransmitters such as glutamate and GABA include contributions from both the vesicular and synaptic compartments, whereas with a longer echo time sequence, vesicular neurotransmitter signals may have dephased to the extent that they no longer contribute significantly to the measured signal. Thus, measurements performed with longer echo time sequences may more accurately reflect changes related to GABAergic synaptic transmission.

Though not statistically significant when adjusted for multiple comparisons, it is worth noting that a negative correlation was observed between Glx OFF and GABA (*r* = −0.56, *p* = 0.07, FDR adjusted *p* = 0.54). This observation is also consistent with previous studies finding that GABA levels correlate negatively with BOLD or a task/stimulus positive condition (Lin et al., 2012; Bednarík et al., 2015; Cleve et al., 2015; Chen et al., 2017; Just and Sonnay, 2017; Mekle et al., 2017) but further suggest a relationship between glutamate and GABA within neural circuits. GABA levels did not show a statistically significant correlation with the FWHM of the water signal, though the differences between Glx signals from the difference and OFF spectra suggest that in a similar fashion, the difference spectrum lacks sufficient spectral information to detect changes with sensitivity comparable to the OFF spectra. Options for measuring GABA in a functional manner at 3 T without spectral editing do exist, such as the STEAM sequence (TE = 20 ms) as used by Kupers et al. (2009) and SPECIAL sequence (TE = 8.5 ms) used by Kühn et al. (2016), both of which were able to measure large, significant changes in GABA in the ACC. In light of the issues raised by this study, they may provide a promising alternative for functional imaging of the BOLD effect and metabolic changes and their use warrants further investigation. However, as stated previously, short echo time sequences may include contributions from vesicular, cytosolic and synaptic GABA, making them less sensitive to compartmental changes in GABA.

The visual stimulation paradigm used in this study was chosen as previous studies have shown it to unambiguously produce a positive BOLD response in the visual cortex, a part of the brain ideal for fMRS experiments as it typically allows placement of a large voxel away from areas of air, bone or fluid that typically contribute to spectral artifacts, and it is typically associated with an increase in glutamate or Glx levels (Mangia et al., 2007; Bednarík et al., 2015; Ip et al., 2017), and decrease in GABA levels (Lin et al., 2012; Mekle et al., 2017). In this study, the MRS voxel was placed in the midline occipital cortex, crossing the longitudinal fissure. It was believed that placing a large voxel in this location would help increase signal-to-noise ratio, and improve spectral quality, however, as can be seen in Figure 4, the positive BOLD signal from an individual participant does not fill the entire voxel. As Just (2020) states, one of the critical issues in determining BOLD responses is positioning of the voxel with respect to the activated area. It is possible that blind placement of the voxel lead to some participants having less positive BOLD signal generated in the MRS voxel. Future studies may benefit from voxel placement guided by fMRI. This was not performed in the present study because it was believed that performing fMRI before MRS could adversely affect the MRS component due to thermal frequency drift.

Taken together, the absence of any statistically significant differences according to the repeated measures *t*-tests suggest that it is possible that no consistent changes were observed between the active and rest conditions due to differences in the amount of activity within the spectroscopy voxel across participants. However, the correlation analysis suggests that for those participants in which there was a haemodynamic response within the spectroscopy voxel that was able to elicit a measurable change in the linewidth of the unsuppressed water peak, there were corresponding metabolic changes. However, this represents only a portion of an already limited sample, and lacks statistical power for reliable conclusions to be drawn.

The main difference between the present study and others in which a change in either Glx or GABA was measured in response to a similar visual stimulation paradigm is firstly that the majority of these studies were conducted using 7 T scanners, and secondly that, with the exception of Ip et al. (2017), participants viewed stimulation in blocks of at least 5 min. The theory behind the shorter, 30 s blocks used in this experiment was that it would be possible to disentangle metabolic changes related to synaptic transmission from changes related to shifts in energy metabolism and possibly the effects of long term potentiation that may be seen with longer stimulation blocks. The absence of any significant measured change in Glx levels between the active and rest blocks in this study suggests that the more consistently measured changes in glutamate or Glx levels measured in other studies represent changes in energy metabolism, but make it difficult to evaluate the performance of the modified MEGA-PRESS technique. It is possible that a longer stimulation block may have elicited a more significant response in the measured Glx levels, however, visual stimulation may not be the optimal form of stimulation for inducing a large, measurable change in neurotransmitter levels.

Mullins (2018) illustrates that with regard to changes in glutamate or Glx in fMRS studies, larger changes were observed in event-related paradigms 13.429% (±3.59) compared to block designs 4.749% (±1.45%) and of those stimuli that elicited a metabolic response, visual stimuli elicited the smallest response, with a mean glutamate increase of 2.318% (±1.227%) with painful stimuli eliciting the largest change 14.458% (±3.736%). This observation holds for fMRS studies into GABA changes as well, with studies using some form of pain stimulus recording ~15% changes in GABA in the anterior cingulate cortex (ACC) (Kupers et al., 2009; Cleve et al., 2017) compared to a ~5% change measured with visual stimulation in the OCC (Mekle et al., 2017). Kühn et al. (2016), in a study measuring changes in the ACC in response to an interference task, namely the Stroop task, measured an 18% increase in GABA in the ACC between the pre-task and task windows. It is possible that the changes in both glutamate and GABA related to synaptic transmission are transient, and that these short-lived changes are diluted when averaging over the entire block. Unfortunately there were too few stimulation blocks per participant for the data to be analyzed as an event-related design. Future studies in evaluating fMRS methods for detecting changes in GABA levels may benefit from implementing event-related or hybrid event-related/block designs where possible.

Finally, one of the fundamental problems facing functional spectroscopy is that of how to disentangle the BOLD effect on spectral linewidth from its consequences on quantitative spectral analysis. It has been established that the BOLD effect affects all peaks in an MR-spectra, not just the unsuppressed water signal, and that this may lead to an overestimation in metabolite levels in quantitative analyses (Zhu and Chen, 2001; Mangia et al., 2006; Bednarík et al., 2015; Ip et al., 2017) and should be corrected for. Creatine is noted for being particularly stable under normal physiological conditions, so much so that metabolite levels are often reported as a ratio relative to creatine (Stagg and Rothman, 2014), a practice that has attracted some criticism as creatine has been shown to be affected in some pathologies as well as being susceptible to the influence of sex hormones (Hjelmervik et al., 2018). Apšvalka et al. (2015) and Mullins et al. (2005) suggest that because no statistically significant change in the concentration of creatine, nor total NAA or choline, was measured in their study, the significant differences in glutamate measured represent a genuine change rather than a generalized effect. Bednarík et al. (2015) and Ip et al. (2017), under the assumption that levels of total creatine should remain stable across changes in neural activity, used the change in the FWHM of the creatine signal between active and rest conditions as a correction factor for the other measured metabolites. Similarly, Mangia et al. (2007) used the difference between spectra acquired during rest and stimulation to calculate a line-broadening correction function to be applied to spectra acquired during the stimulation conditions.

Many of the suggested correction factors for BOLD interference, however, are applied generally and do not account for how the BOLD effect may affect different metabolite signals to different degrees. Zhu and Chen (2001) show the larger, singlet peaks typically found in an MR-spectrum, such as those from creatine, choline and NAA, to be more susceptible to BOLD interference than the smaller multiplets from GABA and Glx. Given the absence of significant correlation between the difference in creatine and choline levels and the change in BOLD signal, there is insufficient evidence to conclude that the BOLD effect has significantly interfered with quantitative analyses. The experimental results are presented as they were measured with the caveat that no correction has been applied and that while significant interference from the BOLD effect appears unlikely it cannot be ruled out entirely.

In conclusion, the modified MEGA-PRESS sequence presented here provides a reliable measure of the BOLD effect through linewidth changes in the unsuppressed water peak, but whether it may also be used to measure associated metabolic changes remains inconclusive. Future studies may benefit from the use of event-related or hybrid block/event-related designs were possible, and the use of sequences that do not rely on spectral editing may be advantageous in light of the increased spectral information and sensitivity they may provide.

## Data Availability Statement

The raw data supporting the conclusions of this article will be made available by the authors, without undue reservation.

## Ethics Statement

The studies involving human participants were reviewed and approved by The Regional Committees for Medical and Health Research Ethics for Western Norway (REK-Vest, REK case number 2016/1629). The patients/participants provided their written informed consent to participate in this study.

## Author Contributions

GD, AC, KH, and RG were involved in the conception and design of the study. AC and LE were involved in programming pulse sequences. GD and AC planned and performed data acquisition with assistance from LE. AC performed spectral analyses. JB and KK performed fMRI analyses. GD conducted statistical analyses and wrote the manuscript with the assistance of KH and RG with critical feedback from all contributing authors. All authors contributed to the article and approved the submitted version.

## Conflict of Interest

AC, LE, KH, and RG have shares in the company NordicNeuroLab A/S which produces add-on equipment for MRI examinations that were used in this study. The remaining authors declare that the research was conducted in the absence of any commercial or financial relationships that could be construed as a potential conflict of interest.
